# Supplementary material for: Hydrophobic-hydrophilic crown-like structure enables aquatic insects to reside effectively beneath the water surface
Source: Commun Biol. 2021 Jun 10;4:708. doi: 10.1038/s42003-021-02228-5 (PMC8192529; doi:10.1038/s42003-021-02228-5)
Supplement: Supplementary file 2 — Supplementary information [file 42003_2021_2228_MOESM2_ESM.pdf]

<u>SUPPORTING INFORMATION</u>

Hydrophobic-hydrophilic crown-like structure enables aquatic insects to reside effectively beneath the water surface

Chiaki Suzuki[1‡], Yasuharu Takaku[1,7‡*], Hiroshi Suzuki[2], Daisuke Ishii[3], Tateo Shimozawa[4], Shuhei Nomura[5], Masatsugu Shimomura[6] and Takahiko Hariyama[1,7*]

[1] Preeminent Medical Photonics Education & Research Center, Institute for NanoSuit Research, Hamamatsu University School of Medicine, 1-20-1 Handayama, Higashi-ku, Hamamatsu 431-3192, Japan

[2] Department of Chemistry, Hamamatsu University School of Medicine, 1-20-1 Handayama, Higashi-ku, Hamamatsu 431-3192, Japan

[3] Life Science and Applied Chemistry, Graduate School of Engineering, Nagoya Institute of Technology, Gokiso-cho, Showa-ku, Nagoya 466-8555, Japan

[4] Research Institute for Electronic Science, Hokkaido University, N21W10, Kita-ku, Sapporo 001-0021, Japan

[5] National Museum of Nature and Science, 4-1-1 Amakubo, Tsukuba 305-0005, Japan

[6] Chitose Institute of Science and Technology, Departments of Bio- and Material Photonics, 758-65 Bibi, Chitose 066-8655, Japan

[7] NanoSuit Inc., 1-20-1 Handayama, Higashi-ku, Hamamatsu 431-3192, Japan

‡ Suzuki C and Takaku Y equally contributed to this work.

*Corresponding Authors:
Takahiko Hariyama (hariyama@hama-med.ac.jp)
Yasuharu Takaku (ytakaku@hama-med.ac.jp)

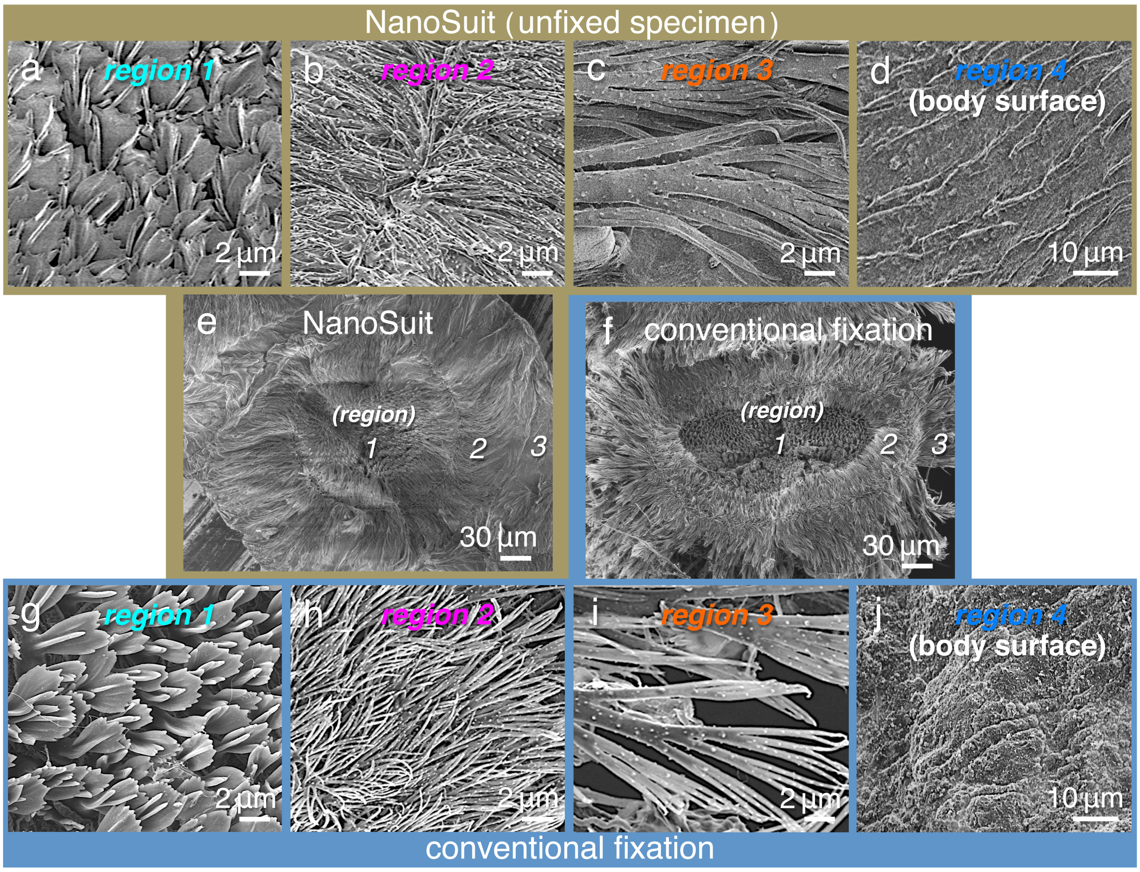

Supplementary Figure 1. Scanning electron micrographs comparing fine structures in the crown treated with the NanoSuit method in **a-e** and specimens prepared by conventional fixation methods for SEM in **f-j**.

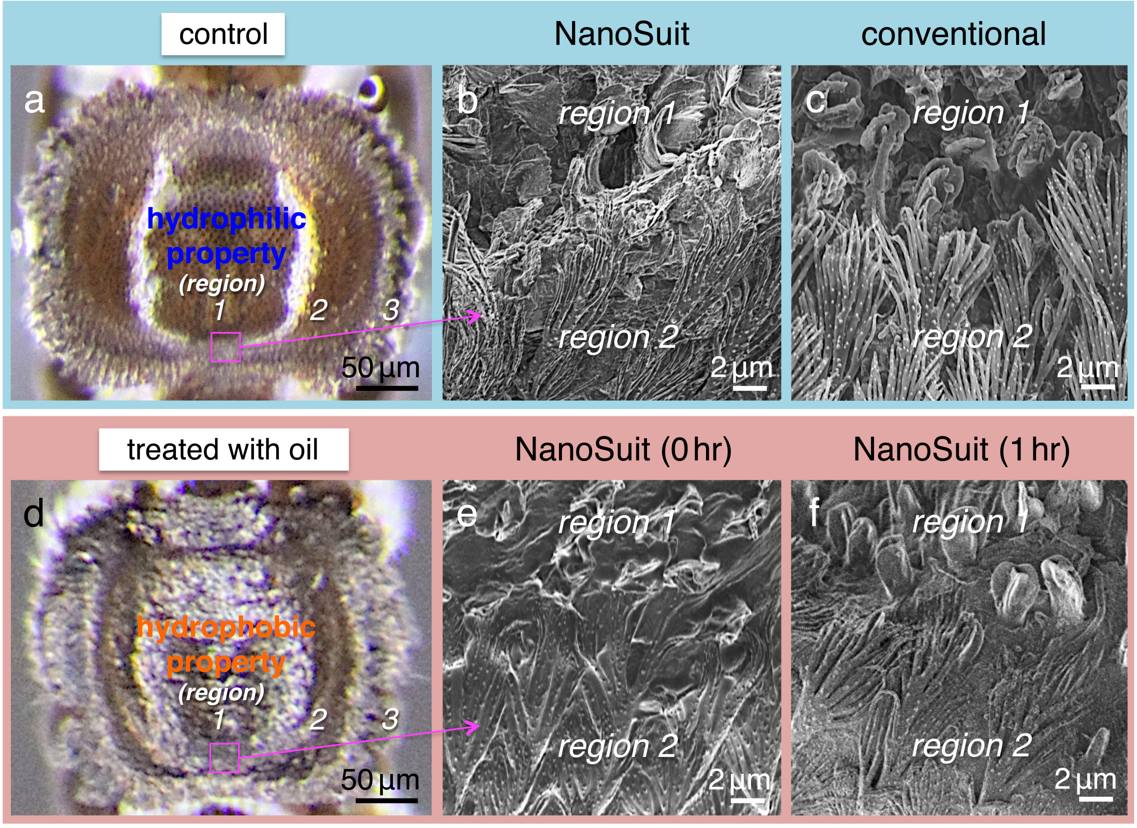

Supplementary Figure 2. Comparison of the control larva in **a-c** and larva in which the crowns were treated with sunflower seed oil in **d-f**. **b**, **c**, **e**, **f** SEM images of areas around the centre structure in the crown. Images of the control larvae were obtained after sample preparation using the NanoSuit method in **b** or by using conventional fixation methods in **c**. Images of the treated larva were obtained after sample preparation using the NanoSuit method, immediately in **e** and 1 h in **f** after the oil treatment.
