# Supplementary material for: Hydrophobic-hydrophilic crown-like structure enables aquatic insects to reside effectively beneath the water surface
Source: Commun Biol. 2021 Jun 10;4:708. doi: 10.1038/s42003-021-02228-5 (PMC8192529; doi:10.1038/s42003-021-02228-5)
Supplement: Supplementary file 3 — Description of Additional Supplementary Files [file 42003_2021_2228_MOESM3_ESM.pdf]

Description of Additional Supplementary Files

File Name: Supplementary Movie 1
Description: Time-lapse side- and/or top-view videos of Dixidae larva with its five unique crown-like structures adhering to the water surface.

File Name: Supplementary Movie 2
Description: Movements of the living larva observed by FE-SEM. The untreated animal was irradiated with an electron beam (1.0 kV) and exposed to high vacuum (10^{-3}-10^{-6} Pa) to observe its fine structure (NanoSuit method).

File Name: Supplementary Movie 3
Description: Time-lapse top-view videos of the larva that was forcefully sunk into water with a pair of forceps.

File Name: Supplementary Movie 4
Description: Time-lapse top-view video images of the tips of the hairy crown structure being re-attached to the water surface after being forcefully sunk into water with a pair of forceps (Fig. 4a, 2.0-7.5 s).

File Name: Supplementary Movie 5
Description: Side-view video of the larva showing spontaneous movements against the water surface. Although the downward force caused by the movements produces curvature in the water surface, the larva is immediately pulled upward to the original water surface by the surface tension.

File Name: Supplementary Movie 6
Description: Side-view video of the larvae captured during the experiment in which the five crowns are pulled down underneath the water surface.

File Name: Supplementary Movie 7
Description: Side-view video of the larvae captured during the experiment in which one crown is pulled down underneath the water surface.

File Name: Supplementary Movie 8
Description: Video capturing the swimming movement of control larvae of *Dixella subobscura*, showing random movement directions. During the movements, *Dixella* larvae showed incomplete contact with the water surface, and occasionally, the bodies of the larvae were perpendicular to the surface.

File Name: Supplementary Movie 9
Description: Video capturing the swimming movement of control larvae of *Dixa longistyla*. The body moves forward, parallel to the water surface.

File Name: Supplementary Movie 10
Description: Video capturing the swimming movement of *Dixa* larvae in which the hydrophilic structures of all five crowns had been treated with hydrophobic sunflower seed oil. The hind segments slip and/or rotate, resulting in a random swimming direction.
